# The Utility of Online Information Sessions for Medical Student Recruitment in Plastic Surgery: A New Paradigm Amidst the COVID-19 Pandemic

**DOI:** 10.1177/22925503211048518

**Published:** 2021-11-24

**Authors:** Yehuda Chocron, Victoria Sebag, Dino Zammit, Stephanie Thibaudeau

**Affiliations:** 1 54473 Division of Plastic and Reconstructive Surgery, McGill University Health Centre, QC, Canada; 2 12367 McGill University, Faculty of Medicine, QC, Canada

**Keywords:** COVID-19, medical education, participation, plastics, social networks, virtual, COVID-19, enseignement de la médecine, participation, plasturgie, réseaux sociaux, virtuel

## Abstract

**Background:** The COVID-19 pandemic has led to increased barriers for medical students seeking to engage with plastic surgery. Traditional approaches such as pursuing clinical electives broadly are no longer feasible and medical students are seeking innovative approaches for engagement. The current study evaluated the efficacy of online information sessions on medical student perception and proposed a timeline for longitudinal medical student recruitment. **Methods:** The McGill Plastic and Reconstructive Surgery residency program held an online information session for medical students focusing on a wide array of topics related to plastic surgery and residency. Following the session, an anonymous survey was sent to participants gauging their satisfaction with the event and potential effects it had on career planning. **Results:** Thirty-four participants completed the survey, comprising more than 60% of annual applicants to Canadian plastic surgery programs. 94% of participants stated that their view of McGill’s training program improved and reported a desire for additional sessions from other training programs. 68% of respondents reported being more likely to consider training at McGill and 100% agreed that such sessions could influence their decision to pursue a given training program. Social media was the most common resource used by participants to gain information on training programs. **Conclusion:** Online information sessions are valuable tools for medical student recruitment and can directly influence their views of a specific training program and affect career planning. Investing in generating high quality content through online forms of communication is paramount as most medical students are turning to these platforms amidst the pandemic.

## Résumé

**Historique**: La pandémie de COVID-19 a accru les obstacles pour les étudiants en médecine qui souhaitent aller en chirurgie plastique. Il n’est plus possible de recourir aux approches classiques, comme la recherche très vaste de stages cliniques, et les étudiants en médecine cherchent des moyens novateurs de procéder. L’étude actuelle a permis d’évaluer l’efficacité des séances d’information en ligne sur la perception des étudiants en médecine et de proposer un calendrier de recrutement longitudinal de ces étudiants en médecine. **Méthodologie**: Le programme de résidence en chirurgie reconstructive et plastique de l’Université McGill a tenu une séance d’information en ligne pour les étudiants en médecine sur un vaste éventail de sujets liés à la chirurgie plastique et à la résidence. Après la séance, les participants ont reçu un sondage anonyme pour évaluer leur satisfaction par rapport à l’événement et les effets potentiels sur leur planification de carrière. **Résultats**: Au total, 34 participants ont rempli le sondage, représentant plus de 60% des candidats annuels aux programmes canadiens en chirurgie plastique. Dans l’ensemble, 94% des participants ont déclaré que leur perception du programme de formation de McGill s’était améliorée et qu’ils souhaitaient participer aux séances d’autres programmes de formation. Par ailleurs, 68% des répondants ont signalé être plus susceptibles d’envisager une formation à McGill et 100% ont convenu que ces séances pouvaient influer sur leur décision d’opter pour un programme de formation donné. Les réseaux sociaux étaient la principale ressource qu’utilisaient les participants pour obtenir de l’information sur les programmes de formation. **Conclusion**: Les séances d’information en ligne sont des outils précieux pour recruter des étudiants en médecine, peuvent avoir une influence directe sur leurs points de vue au sujet d’un programme de formation donné et peuvent se répercuter sur leur planification de carrière. Il est essentiel d’investir dans la production de contenu de qualité transmis par diverses formes de communications en ligne, car la plupart des étudiants en médecine se tournent vers ces plateformes en cette période de pandémie.

## Introduction

The COVID-19 pandemic has had significant impacts on medical student education and career planning due to reduced clinical exposure.^1,2^ The public health initiatives put into place have prevented medical students from pursuing clinical electives outside of their respective institutions which has limited their ability to explore training programs. Plastic surgery has typically been a field that is underrepresented in medical school curriculum making it difficult for medical students to gain clinical exposure early on when considering career paths.^3^ Given the added barrier of the COVID-19 pandemic, opportunities have become extremely scarce for medical students pursuing plastic surgery. Although medical students are likely seeking innovative ways to engage with plastic surgery amidst the pandemic, training programs will play a critical role in targeting prospective students. Ultimately, medical students will serve as the future leaders of plastic surgery and it is therefore crucial for training programs to invest in engaging with these future leaders throughout this period.

Plastic surgeons are always on the forefront of innovation and training programs are at a period of adaptation in order to find innovative ways to connect with medical students. These initiatives have included social media campaigns through online presence, connecting with residents and program directors through online information sessions and invitations to remote teaching gatherings.^4^ Although many online learning resources have been made available to medical students, and the call for the importance of online mentorship continues to be explored, there is a lack of literature explicitly evaluating the effect of online information sessions on specialty choice or specific training program for prospective medical students.^5^ The overarching goal of the current study aims to evaluate the utility of online information sessions and the effects they may have on career planning for senior level medical students. This was accomplished by assessing medical students' perception of an online information session held by the McGill University Plastic and Reconstructive Surgery training program. The results from this study will serve as a proof of concept on the utility of these sessions for training programs seeking to increase their engagement with prospective medical student applicants. Furthermore, a time-line based engagement curriculum with plastic surgery programs and medical students is proposed based on year of study.

## Methods

### Information Session

In September 2020, the McGill University Plastic and Reconstructive Surgery (PRS) residency program held an online information session for medical students interested in learning about the residency training program. Social media posts and announcements on the program website were used to inform medical students. The session was hosted by the program director (senior author; ST) and a team comprised of junior and senior residents. The session covered an array of topics including program structure, clinical exposure, research opportunities, mentorship and the admissions process. The formal presentation was followed by a question and answer period allowing medical students to discuss any queries with the resident team.

### Survey

Following the information session, an anonymous web-based survey was sent to participants using Google Forms™ (see PDF, Supplemental Digital Content 1, the complete survey). The survey was sent out in November 2020 followed by two reminder emails within a five-week period. Participation was completely anonymous and respondents were able to withdraw from completing the survey at any point without prejudice. The results were pooled across all participants for descriptive purposes and presented in table or graphic form for visual purposes.

## Results

### Demographics

Overall, 34/40 (85%) participants that attended the online information session completed the survey, which consisted of our study population. Participants included medical students from eight Canadian provinces with Quebec (n = 11/34, 32%) and Ontario (n = 11/34, 32%) being most common (Table 1). Overall, 53% (n = 18/34) of participants were female and 47% (n = 16/34) were male. Additionally, 74% of attendees (n = 25/34) were fourth year medical students and 12% (n = 4/34) were in their third year of medical school. In terms of motivation for attending the session, 88% of participants (n = 30/34) reported already having decided to pursue a career in plastic surgery while 12% (n = 4/34) were considering plastic surgery as a career choice.

**Table 1.** Provincial breakdown of participants

Province	N	%
Quebec	11	33%
Ontario	11	33%
Alberta	3	9%
British Columbia	3	9%
Manitoba	2	6%
Saskatchewan	1	3%
PEI	1	3%
Nova Scotia	1	3%
**Total**	**33**	**100%**

### Use of Social Media:

Social media played an important role in both the recruitment to the information session, as well as program information dissemination. The majority of participants (n = 28/34, 82%) were informed of the event through social media posts. Other avenues included directly contacting the program (n = 3/34, 8.8%) or residents (n = 1/34, 3%). Furthermore, the most common resource used by respondents to gain information on specific programs was social media (n = 18/34, 53%) followed by program websites (n = 10/34, 29%) and directly contacting residents (n = 6/34, 18%).

### Impact of Covid-19 on Exposure:

**Figure 1.**
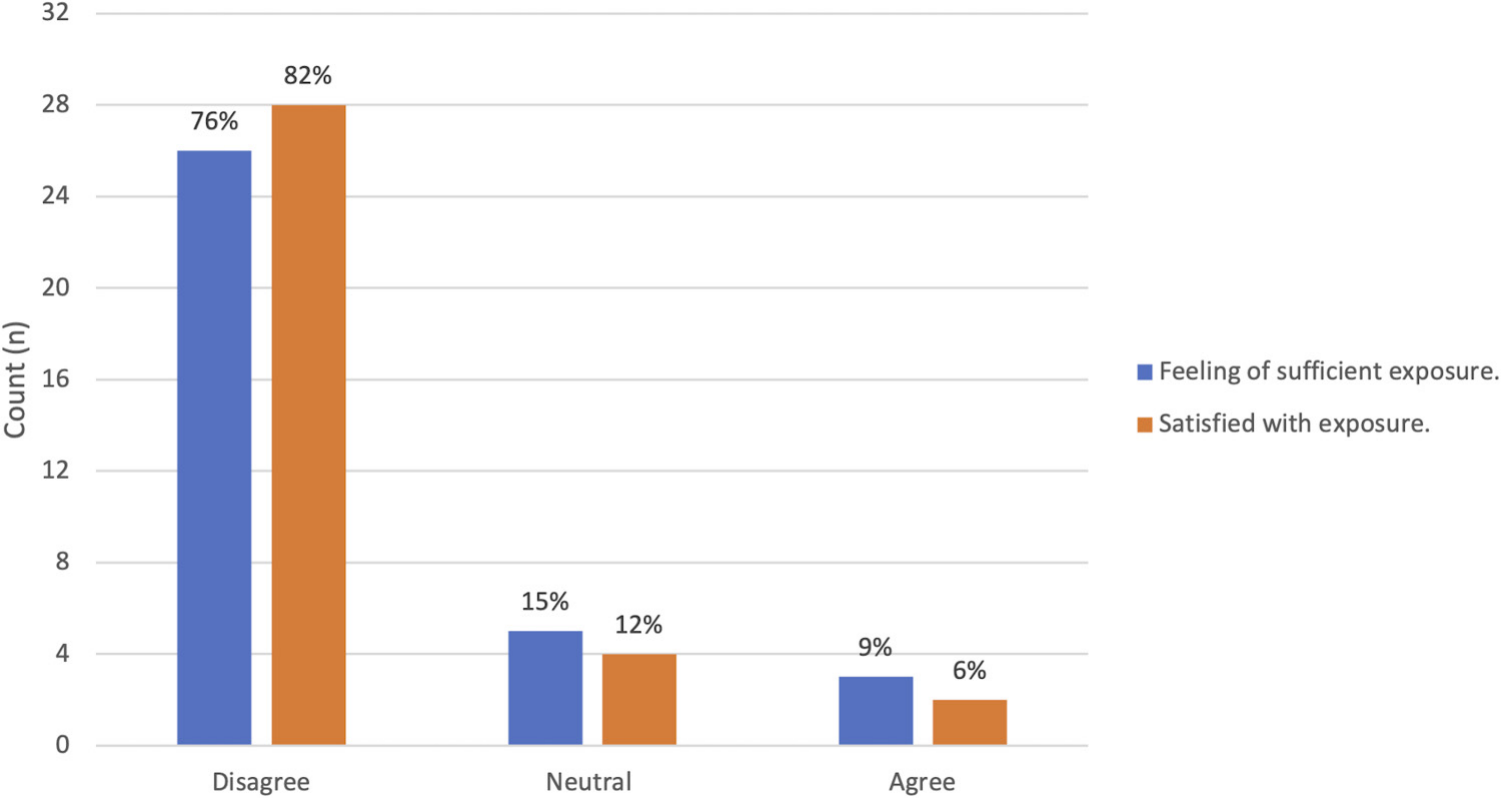
Participant level of satisfaction with regards to plastic surgery exposure during the pandemic

**Figure 2.**
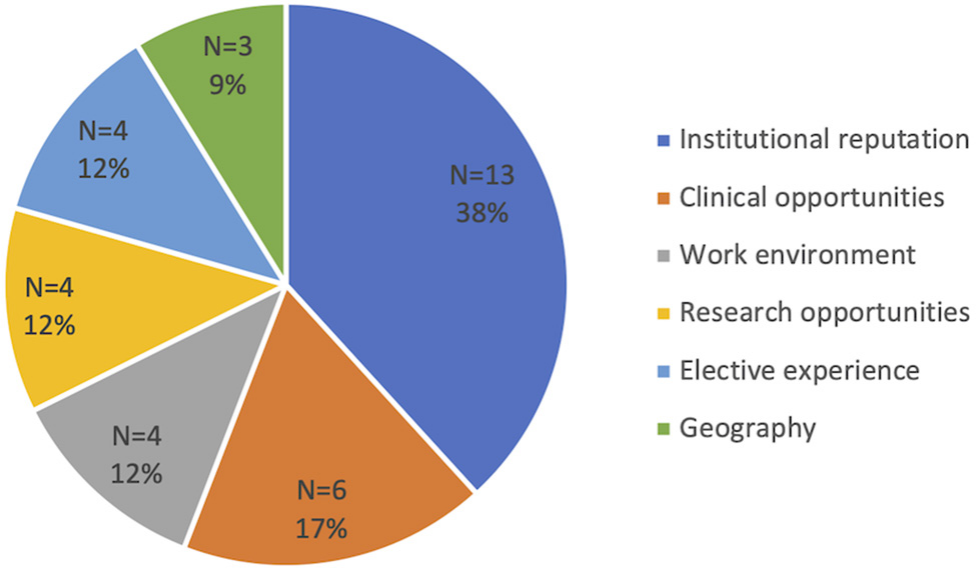
Factors guiding residency program selection

Overall, 76% of respondents (n = 26/34) reported having insufficient exposure to plastic surgery throughout the pandemic and only 6% of respondents (n = 2/34) reported being satisfied with their exposure (**Figure 1)**. When asked about critical factors that impact medical student's selection of a residency program in the absence of pursing a plastic surgery elective, “institutional reputation” (n = 13/34, 38.2%), and “clinical opportunities” (n = 6/34, 17.6%) were the most common cited factors (**Figure 2)**.

### Benefit and Effects on Medical Student Perception

**Figure 3.**
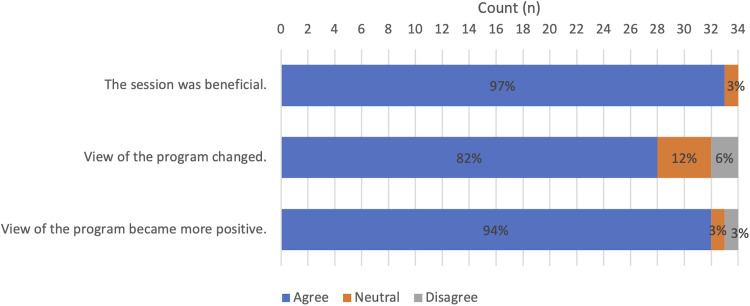
Effect of online information session on medical student perception

**Figure 4.**
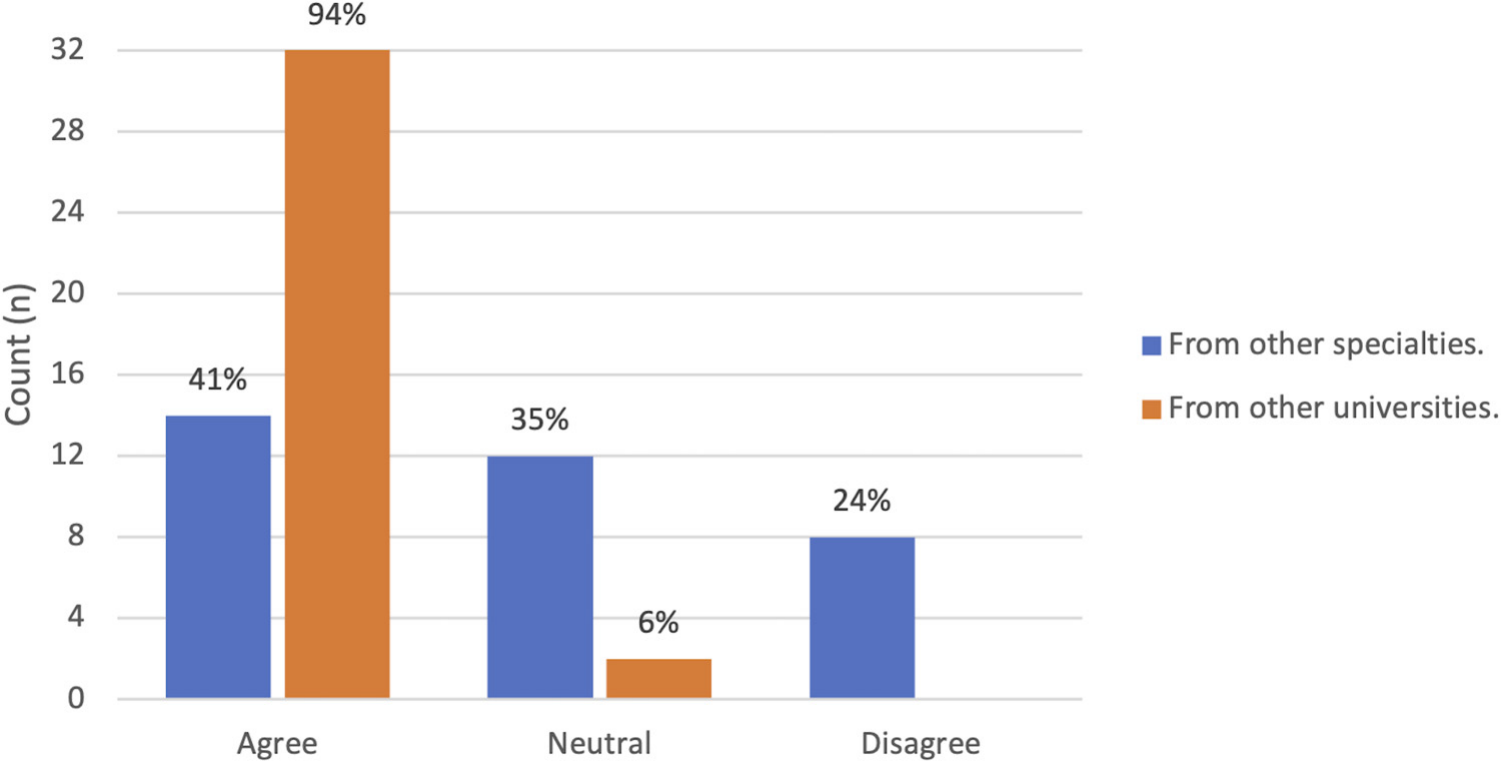
Student demand for online information sessions

**Figure 5.**
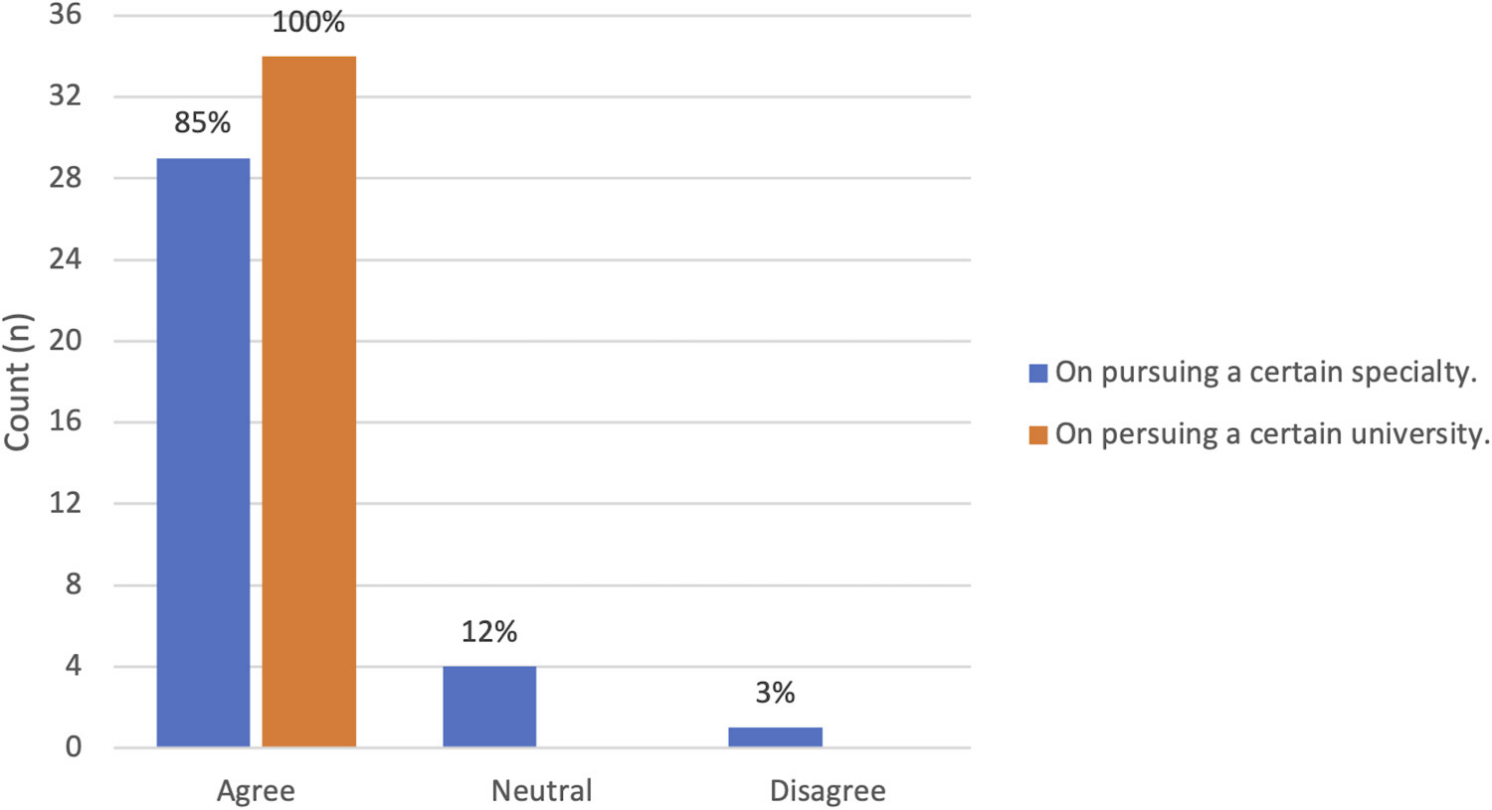
Influence of information session on medical student career planning

Overall, 97% of participants (n = 33/34) reported that the information session was beneficial to their knowledge of the McGill University plastic surgery program, whereas 82% of respondents (n = 28/34) reported that their perception of the McGill University plastic surgery program changed. Additionally, 94% (n = 32/34) reported that their view of the program became more positive following the session (**Figure 3)**. In terms of effects on career planning, 79% (n = 27/34) reported already having decided to pursue a career in plastic surgery, with 68% of respondents (n = 23/34) reported being more likely to consider training at McGill University following the session (as opposed to 26% n = 9/34 who were already certain that they would consider our program). Overall, 94% (n = 32/34) would appreciate additional sessions in plastic surgery from other universities (**Figure 4).** Finally, 85% of respondents (n = 29/34) reported that online information sessions could influence their decision to pursue a given specialty and 100% (n = 34/34) agreed that it could influence their decision to pursue a specific plastic surgery training program (**Figure 5)**.

## Discussion

Given the ongoing COVID-19 pandemic and the limited contact between medical students and training programs, avenues for communication and engagement are crucial. The purpose of the current study was to assess the effect of online information sessions on medical student perception and career planning. Using McGill Plastic and Reconstructive Surgery's online information session as a model, it was determined that online information sessions are extremely valuable in educating prospective medical students and can directly impact their decision to pursue a specific program or specialty. Overall, twenty-five fourth year medical students responded to the survey, which constitutes 49% of annual American and Canadian applicants to Canadian plastic surgery programs, indicating that our results represent the collective view of annual plastic surgery applicants.^6^ As expected, there is an increasing demand for online information sessions amidst the pandemic as 94% of respondents reported a desire for additional sessions from other Plastic Surgery programs. Our results mirror the current societal changes in communication, with most medical students turning to online social media platforms to obtain information rather than the traditional direct routes of communication with the program. Moreover, online information sessions can directly alter the perception of medical students and can increase their likelihood of pursuing specific training programs as is shown in the current study.

The factors that influence residency selection in medical students have been studied and include mentorship, elective experience and research opportunities, which are concurrent with the findings in this study.^7,8^ A recent study assessing modalities for medical student recruitment in Otolaryngology amidst the pandemic suggested that virtual electives and weekly mentorship meetings seem to be a promising avenue to engage medical students within specialties.^9^ In a more recent study by Tanaka et al. online information sessions were used to increase engagement with applicants throughout the pandemic, and the sessions proved to be beneficial, in keeping with our data.^10^ Anderi et al. assessed general student engagement throughout COVID-19 and found that medical students are seeking opportunities to engage with healthcare professionals through online platforms, further revealing the need for such sessions.^11^ Additionally, a study by Byrnes et al. found that only 20% of surveyed medical students reported that the COVID-19 pandemic would alter their first choice in residency selection. Due to the inability to explore multiple programs with restrictions in clinical electives, students believed that it would be difficult to gain sufficient exposure in other institutions and secure recommendations from attending physicians.^12^ This highlights the fear that students currently have regarding their inability to explore training programs and interact with faculty members outside of their host institution. This can potentially effect their rank order list during the admission process as they may not be confident in the impression they have on residency programs outside of their home institution. Recently, Go et al. reported on the importance of virtual teaching for residency program selection among medical students. Although our data was consistent with the effects it can have on the perception of a specific training program, the majority of respondents in the current study were already decided on plastic surgery and were in their final year of medical school.^9^ It is therefore no surprise that decided students in their final year are most likely to attend these sessions and are seeking opportunities to engage with training programs in their desired specialty rather than exploring other disciplines. Therefore, programs could consider hosting introductory sessions to their specialty for students in earlier years of medical school while hosting formal sessions on residency program structure for senior medical students.

**Figure 6.**
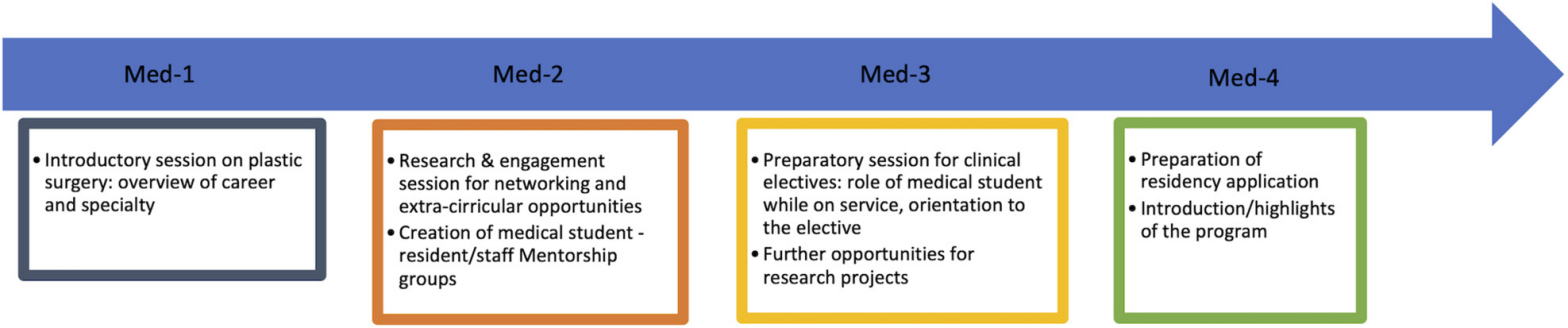
Proposed timeline for medical student recruitment and engagement sessions

Currently, medical students across Canada are dissatisfied with their exposure to plastic surgery amidst the pandemic. Although opportunities for clinical exposure and abroad electives are largely dictated by public health guidelines, there is a clear need to increase social and academic activities with medical students, with formal online interactive sessions being a useful tool for engagement. In keeping with the lack of direct contact with medical students, the majority of prospective applicants are turning to social media as the predominant resource to gain information on training programs amidst the pandemic. Social media content is the likely becoming the applicants “first impression” of a specific training program and can therefore alter their perception. In the COVID-19 era, it is likely more critical for training programs to invest in developing safe and ethically modulated social media presence to be able to reach medical students broadly. We therefore propose having multiple sessions throughout the year targeting medical student of different levels. As an example, sessions targeting first year medical students would focus more on an overview of plastic surgery as a field, whereas sessions engaging with fourth year students would be focused on presenting highlights of the presenter's plastic surgery training program (Figure 6).

Overall, our study exposed a current reality for medical students that programs would benefit from understanding and adapting to. They appreciate online information sessions, and confirm that these sessions can greatly influence applicant choices for institutions in lieu of actually being immersed in the programs while electives are restricted. Online information sessions can also offer programs the ability to engage with medical students that may have otherwise been unable to secure clinical electives. This can be accomplished by employing smaller “breakout sessions” offering faculty members the ability to directly interact with medical students. The importance of social media has also become apparent and establishing a strong online presence (social media, blogs, active program websites) is critical to maintain active engagement with prospective applicants.

This study was limited in that only 40 medical students attended the session and 34 responded to our survey (25 of which were in their fourth year). However, according to the Canadian Federation of Medical Students (CFMS), the 5-year average of yearly plastic surgery applicants (both American and Canadian) is 496. Therefore, by virtue of our sample size, we likely captured over 50% of applicants, representing eight Canadian provinces, making our results an accurate depiction of current medical students interested in Canadian plastic surgery training programs. We cannot exclude the possibility that responder bias may have affected our results, although unlikely given the anonymity of the survey. In addition, the survey was composed of multiple-choice/pre-written answer options, which may have limited our ability to capture the exact thoughts of participants, their stressors and overall feedback; however, the construct of our questions and answers was done so that trends could be quantitatively analyzed and compared. This was also done in the interest of making the survey quick for busy students to answer without in-depth expansion on their answers, which limits our data on their preferences in terms of session organization and topics. However, our goal was to assess the impact and efficacy of these sessions, which our survey successfully accomplished.

## Summary of Recommendations

In terms of the specific structure for virtual sessions, ours was divided into a formal presentation offered by the program director and residents followed by an informal question and answer period. Given that it was our first virtual event, the presentation portion was broad and focused on program structure, admissions process, opportunities for clinical and research involvement and networking. This was followed by a question and answer period which was well received by medical students from various years. Given that our study revealed that institutional reputation and clinical opportunities are the most important factors for applicants when considering residency programs, we recommend highlighting these points throughout the presentation. In the future, we recommend breaking down sessions according to medical student level and our proposed recruitment schedule (Figure 6) can serve as an outline for programs to incorporate theses virtual events throughout the year. Finally, we suggest that these sessions be hosted regardless of pandemic restrictions as many factors may influence a student's ability to secure visiting electives, including finances and the elective specialty cap which all Canadian medical students must respect. By using social media presence, website presence, and virtual communication, students can always learn about and access resources to develop their interest in plastic surgery programs.

## Conclusion

Medical students represent the future of plastic surgery and ensuring that they are properly exposed to programs as they make choices about their future careers is beneficial to both students, the programs they may join, and their future patients. The COVID-19 pandemic was the thrust required for many training programs to shift their communication methodology to online and social media platforms. Our study has demonstrated that it is possible to adapt quickly to the restrictions imposed by the COVID-19 pandemic. Through online orientation sessions, we can positively influence students and make them more confident exploring various training programs. Ultimately, given that the nature of a competitive residency program like plastic surgery is indeed a “seller's market”, programs have the luxury of selection and can likely attract the same number of applicants without investing in expensive recruitment methods. In this case, alternative avenues such as virtual recruitment sessions as is shown by the current study, serve as useful alternatives to the pre-pandemic standard. Further investigations as the pandemic continues to unfold, affecting education and residency recruitment should assess the impact of online exposure to programs on the volume of program applicants and overall success of match.

*Google and Google Forms are trademarks of Google LLC and this research is not endorsed by or affiliated with Google in any way.

## Supplemental Material

sj-docx-1-psg-10.1177_22925503211048518 - Supplemental material for The Utility of Online Information Sessions for Medical Student Recruitment in Plastic Surgery: A New Paradigm Amidst the COVID-19 Pandemic Click here for additional data file. Supplemental material, sj-docx-1-psg-10.1177_22925503211048518 for The Utility of Online Information Sessions for Medical Student Recruitment in Plastic Surgery: A New Paradigm Amidst the COVID-19 Pandemic by Yehuda Chocron, Victoria Sebag and Dino Zammit, Stephanie Thibaudeau in Plastic Surgery

## References

[1] Ferrel MN Ryan JJ . The impact of COVID-19 on medical education. Cureus. Mar. 31 2020;12(3):e7492. doi:10.7759/cureus.7492 10.7759/cureus.7492 PMC7193226 32368424

[2] Franchi T . The impact of the covid-19 pandemic on current anatomy education and future careers: a Student's Perspective. Anat Sci Educ. May 2020;13(3):312-315. doi:10.1002/ase.1966 32301588 10.1002/ase.1966 PMC7262338

[3] Farid M Vaughan R Thomas S . Plastic surgery inclusion in the undergraduate medical curriculum: perception, challenges, and career choice-A comparative study. Plast Surg Int. 2017;2017(1):9458741. doi:10.1155/2017/9458741 28630768 10.1155/2017/9458741 PMC5463111

[4] Cho MJ Hong JP . The emergence of virtual education during the COVID-19 pandemic: the past, present, and future of the plastic surgery education. J Plast Reconstr Aesthet Surg. Jan. 10 2021;74(6):1413-1421. doi:10.1016/j.bjps.2020.12.099 10.1016/j.bjps.2020.12.099 PMC7797168 33541826

[5] Abdelhamid K ElHawary H Gorgy A Alexander N . Mentorship resuscitation during the COVID-19 pandemic. AEM Educ Train. Sep. 26 2020;5(1):132-134. doi:10.1002/aet2.10538 10.1002/aet2.10538 PMC7537243 33043229

[6] Canadian Federation of Medical Students. MatchStats: A Discipline By Discipline Briefing of CaRMS Match and Electives Data. 2018. https://www.cfms.org/files/matchbook/MatchStats.pdf.

[7] McCloskey CB Johnson K Brissette M , et al. Factors influencing US allopathic medical students to choose pathology as a specialty. Acad Pathol. Jan. Dec 2020;7(2374289520951924). doi:10.1177/2374289520951924 10.1177/2374289520951924 PMC7557358 33110939

[8] Janis JE Barker JC . Medical student mentorship in plastic surgery: the Mentor's Perspective. Plast Reconstr Surg. Nov 2016;138(5):925e-935e. doi:10.1097/PRS.0000000000002670 10.1097/PRS.0000000000002670 27783013

[9] Go B Rajasekaran K . Effect of COVID-19 in selecting otolaryngology as a specialty. Head Neck. Jul 2020;42(7):1409-1410. doi:10.1002/hed.26251 32633093 10.1002/hed.26251 PMC7272945

[10] Tanaka ME Brideau HR An TJ McLoud TC Little BP Kelly HR . Utilization of a virtual information session to increase engagement With prospective applicants in the setting of COVID-19. Curr Probl Diagn Radiol. Nov. 15 2020;50(3):351-355. doi:10.1067/j.cpradiol.2020.11.005 10.1067/j.cpradiol.2020.11.005 PMC9759597 33257095

[11] Anderi E Sherman L Saymuah S Ayers E Kromrei HT . Learning communities engage medical students: a COVID-19 virtual conversation series. Cureus. Aug. 6 2020;12(8):e9593. doi:10.7759/cureus.9593 10.7759/cureus.9593 PMC7478637 32923199

[12] Byrnes YM Civantos AM Go BC McWilliams TL Rajasekaran K . Effect of the COVID-19 pandemic on medical student career perceptions: a national survey study. Med Educ Online. Dec 2020;25(1):1798088. doi:10.1080/10872981.2020.1798088 32706306 10.1080/10872981.2020.1798088 PMC7482653

