# Supplementary material for: The Utility of Online Information Sessions for Medical Student Recruitment in Plastic Surgery: A New Paradigm Amidst the COVID-19 Pandemic
Source: Plast Surg (Oakv). 2021 Nov 24;31(3):293–9. doi: 10.1177/22925503211048518 (PMC10467434; doi:10.1177/22925503211048518)
Supplement: sj-docx-1-psg-10.1177_22925503211048518 - Supplemental material for The Utility of Online Information Sessions for Medical Student Recruitment in Plastic Surgery: A New Paradigm Amidst the COVID-19 Pandemic [file sj-docx-1-psg-10.1177_22925503211048518.docx]

Online Information Session Benefits

Please answer the following questions regarding your attendance to McGill University's online plastic surgery program information session (September 30th, 2020).

This information will allow us to examine the potential benefits of hosting online information sessions for both the university and the residency program.

1. What is your current level in medicine?

*Mark only one circle.*

- Med-1
- Med-2
- Med-3
- Med-4
- N/A

2. Which province is your medical school located in?

*Mark only one circle.*

- Alberta
- British Columbia
- Manitoba
- New Brunswick
- Newfoundland and Labrador
- Nova Scotia
- Ontario
- PEI
- Quebec
- Saskatchewan

3. What is your preferred gender?

*Mark only one circle.*

- Female
- Male
- Non-Binary
- Other/Prefer not to say

4. What factor is most important to you when considering a residency program?

*Mark only one circle.*

- Geography
- Clinical opportunities
- Research opportunities
- Elective experience
- Institutional reputation
- Work environment
- Established mentorship

5. What resources have you been using to learn about programs amidst the COVID-19 pandemic?

*Mark only one circle.*

- Program website
- Contacting program director
- Contacting residents
- Contacting medical students
- Social media

6. What prompted you to join the event?

*Mark only one circle.*

- Already decided on plastic surgery
- Considering plastic surgery
- Interested in surgical specialties
- Still undecided on specialty

7. How did you hear about the event?

*Mark only one circle.*

- Social media posts
- Announcement on program website
- Word of mouth
- Contacting program
- Other:

Please answer whether you agree with the following statements:

8. Since the COVID-19 pandemic began, I have had sufficient exposure to plastic surgery.

*Mark only one circle.*

- Strongly agree
- Agree
- Neutral
- Disagree
- Strongly disagree

9. I am satisfied with my exposure to plastic surgery since the COVID-19 pandemic.

*Mark only one circle.*

- Strongly agree
- Agree
- Neutral
- Disagree
- Strongly disagree

10. The online information session was beneficial to my knowledge of McGill’s plastic surgery program.

*Mark only one circle.*

- Strongly agree
- Agree
- Neutral
- Disagree
- Strongly disagree

11. My view on McGill’s plastic surgery program changed with the online information session.

*Mark only one circle.*

- Strongly agree
- Agree
- Neutral
- Disagree
- Strongly disagree

12. My view on McGill’s plastic surgery program became more positive with the online information session.

*Mark only one circle.*

- Strongly agree
- Agree
- Neutral
- Disagree
- Strongly disagree

13. I am more likely to pursue plastic surgery after this session.

*Mark only one circle.*

- Strongly Agree
- Agree
- No change: I was already sure I would pursue plastic surgery
- No change: I was already sure I would not pursue plastic surgery
- Disagree
- Strongly disagree

14. I am more likely to consider McGill University for my residency after this session.

*Mark only one circle.*

- Strongly Agree
- Agree
- No change: I was already considering McGill
- No change: I already knew I did not want to go to McGill
- Disagree
- Strongly disagree

15. I would appreciate more sessions like these, in other specialties.

*Mark only one circle.*

- Strongly agree
- Agree
- Neutral
- Disagree
- Strongly disagree

16. I would appreciate more sessions like these, from other universities.

*Mark only one circle.*

- Strongly agree
- Agree
- Neutral
- Disagree
- Strongly disagree

17. These sessions could influence my decision to pursue a certain specialty.

*Mark only one circle.*

- Strongly agree
- Agree
- Neutral
- Disagree
- Strongly disagree

18. These sessions could influence my decision to attend a certain university.

*Mark only one circle.*

- Strongly agree
- Agree
- Neutral
- Disagree
- Strongly disagree
